# Factors Related to Unemployment in Europe. A Cross-Sectional Study from the COURAGE Survey in Finland, Poland and Spain

**DOI:** 10.3390/ijerph15040722

**Published:** 2018-04-11

**Authors:** Matilde Leonardi, Davide Guido, Rui Quintas, Fabiola Silvaggi, Erika Guastafierro, Andrea Martinuzzi, Somnath Chatterji, Seppo Koskinen, Beata Tobiasz-Adamczyk, Josep Maria Haro, Maria Cabello, Alberto Raggi

**Affiliations:** 1 Neurological Institute C. Besta IRCCS Foundation, Neurology, Public Health and Disability Unit, 20133 Milan, Italy; davide.guido@istituto-besta.it (D.G.); rui.quintas@istituto-besta.it (R.Q.); fabiola.silvaggi@istituto-besta.it (F.S.); erika.guastafierro@istituto-besta.it (E.G.); alberto.raggi@istituto-besta.it (A.R.); 2 Neurological Institute C. Besta IRCCS Foundation, Clinical and Experimental Epileptology Unit, 20133 Milan, Italy; 3 E. Medea Scientific Institute, Conegliano-Pieve di Soligo Research Centre, 31015 Conegliano Veneto, Italy; andrea.martinuzzi@lanostrafamiglia.it; 4 Information, Evidence and Research Unit, World Health Organization, 1211 Geneva, Switzerland; chatterjis@who.int; 5 Ageing, Disability and Functioning Unit, National Institute for Health and Welfare, 00271 Helsinki, Finland; seppo.koskinen@thl.fi; 6 Department of Medical Sociology, Jagiellonian University Medical College, 31-034 Krakow, Poland; mytobias@cyf-kr.edu.pl; 7 Parc Sanitari Sant Joan de Déu, University of Barcelona, CIBERSAM, 08830 Barcelona, Spain; jmharo@pssjd.org; 8 Instituto de Salud Carlos III, Centro de Investigación Biomédica en Red de Salud Mental, CIBERSAM, 28029 Madrid, Spain; maria.cabello@uam.es; 9 Department of Psychiatry, Universidad Autonoma de Madrid, 28029 Madrid, Spain

**Keywords:** unemployment, self-rated health, alcohol consumption, survey data, physical activity, healthy behaviors, depression, model selection

## Abstract

*Background*: Research addressing the impact of a large number of factors on unemployment is scarce. We aimed to comprehensively identify factors related to unemployment in a sample of persons aged 18–64 from Finland, Poland and Spain. *Methods*: In this cross-sectional study, factors from different areas were considered: socio-demographic indicators, health habits, chronic conditions, health state markers, vision and hearing indicators, and social networks and built environment scores. *Results*: Complete data were available for 5003 participants, mean age 48.1 (SD 11.5), 45.4% males. The most important factors connected to unemployment were health status indicators such as physical disability (OR = 2.944), self-rated health (OR = 2.629), inpatient care (OR = 1.980), and difficulties with getting to the toilet (OR = 2.040), while the most relevant factor related to employment were moderate alcohol consumption (OR = 0.732 for non-heavy drinkers; OR = 0.573 for infrequent heavy drinkers), and being married (OR = 0.734), or having been married (OR = 0.584). Other factors that played a significant role included presence of depression (OR = 1.384) and difficulties with near vision (OR = 1.584) and conversation hearing (OR = 1.597). *Conclusions*: Our results highlight the importance of selected factors related to unemployment, and suggest public health indications that could support concrete actions on modifiable factors, such as those aimed to promote physical activity and healthy behaviors, tackling depression or promoting education, in particular for the younger.

## 1. Introduction

Unemployment represents a major social problem as it determines loss of income, increases the risk of poverty and may affect overall health [1,2]. In fact, being employed is a source of financial security, provides people the opportunity to fulfil a social and family role, and is important for physical and mental health [3,4]. Unemployment means a change in social position, particularly in the family, and is usually perceived as a very stressful life event [5,6,7] with relevant consequences for health [8,9]. Precise and commonly shared definitions of employment and unemployment status are lacking and different studies addressed them differently: these include long-term vs. short-term unemployment, or temporary interruptions, and are referred to people of working age (but not student or in training) that are actively looking for work. The definition of employment given by the International Labour Organization comprises people that, in a given period of time (e.g., the previous week) have been without work and are currently available for working and actively seeking a job [10]. Such a definition, however, leaves out all those persons that have not been working and are not seeking a job for different reasons, e.g., house workers or those that are discouraged from the possibility to get a job because of an economic crisis. Thus, employment and unemployment conditions are complex and have a dynamic nature and this variability must be considered [11].

Several studies focused on the effects of unemployment on health, as a recent literature review has shown [3,12], and many studies focus on the effects of a specific health condition, and of its outcomes in terms of disability, on working capacity and/or employment status or, in other words, on the cost associated to different diseases [13,14,15,16,17]. However, employment status is affected by several variables, including non-health related ones. Among them, education, age and gender are likely to be the most relevant ones. Low education is an important determinant of unemployment status [18,19,20] and, by interacting with poor health, determines an exacerbation of its effect [21,22]. There is also an age-related effect, with younger people facing more difficulties with entering and remaining in the labor market if they have lower education and poor health [23,24,25]. Finally, literature shows that a relevant gender gap exists with regard to employment status, with men having considerably higher employment rates than women [26,27,28,29].

Studies addressing the relationship between unemployment and health relied on variables that act as adjusting (potentially confounding) variables and are not presented with stratified estimates for each group (e.g., by gender, marital status, age, etc.) [12], and the effects of health on employment status (and vice versa) is usually different across groups. The most disadvantaged groups vary, depending on the target population and on the historical period in which the study was carried out: in fact, the 2007 economic crisis has led to profound differences in the composition of unemployed groups, for example by reducing the gender gap [30]. For all of the above reasons, understanding the factors associated to unemployment is of crucial importance, but research addressing the impact of a large number of factors on unemployment is almost lacking.

The aim of this paper is to comprehensively identify the potential factors contributing to unemployment in a large European population study sample of persons aged 18–64 that were enrolled by the Framework Programme 7 Collaborative Research on Ageing in Europe (COURAGE in Europe) project. We analyzed risks and protective factors for unemployment among a wide set of candidate regressors available from COURAGE in Europe Project, such as demographic data, chronic conditions, health and health habits information, social networks and built environment variables.

## 2. Materials and Methods

### 2.1. Study Design and Sample

The COURAGE in Europe project is a cross-sectional survey that interviewed nationally representative samples of the general non-institutionalized adult population from three European countries: Finland, Poland, and Spain. These countries were selected to give a broad representation spanning different European regions, taking into consideration their population and health characteristics. The surveys were conducted between May 2011 and March 2012.

**Figure 1:**
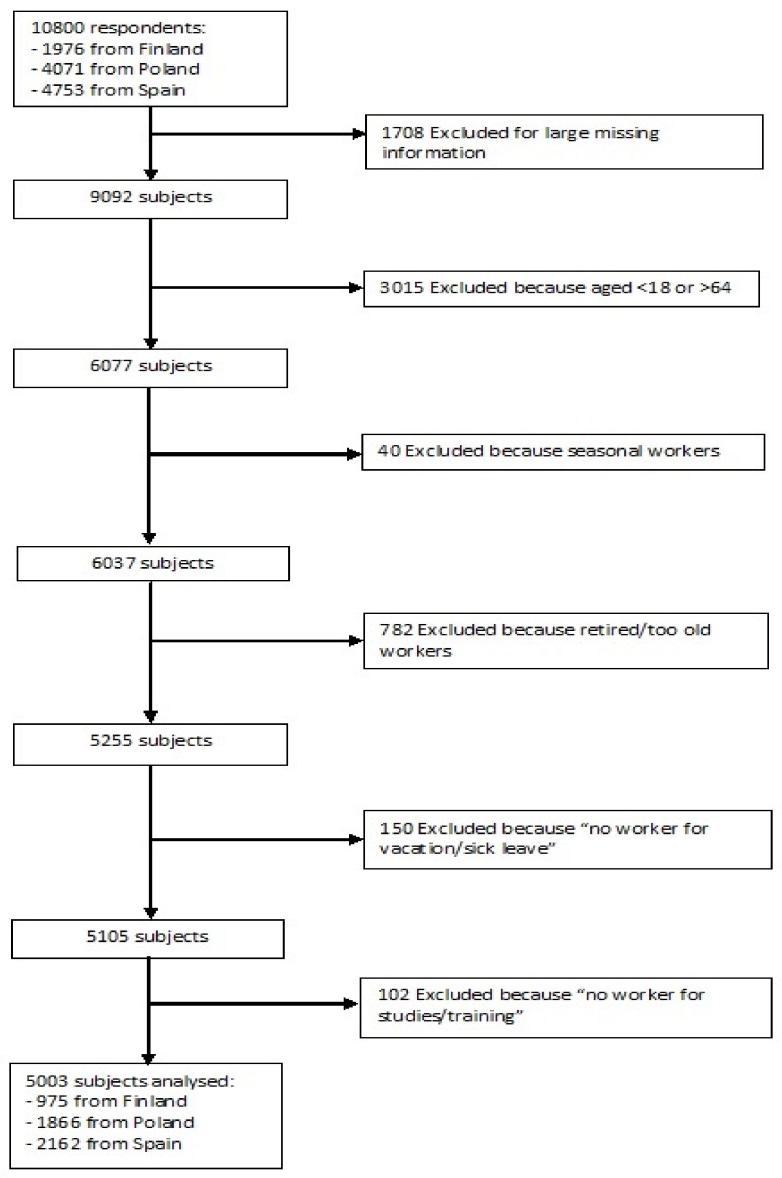
Flow diagram of the sample selection.

The whole sample comprised 10,800 respondents: 1976 from Finland (response rate 53.5%), 4071 from Poland (response rate 66.5%), and 4753 from Spain (response rate 69.9%) [31]. From the whole sample, a sub-sample subjects aged 18–64 years old, i.e., people of working age, was considered. The unemployed outcome was defined by dichotomous question “Have you worked for at least 2 days during the last 7 days?” Between “no” responses, exclusions were performed for four categories of respondents: seasonal workers (*n* = 40), retired respondents (*n* = 782), non-workers for “vacation/sick leave” (*n* = 150) and, finally, students (*n* = 102). Therefore, the group of unemployed people consisted of those actively looking for a job and available to start it and of house workers. Finally, 1708 respondents were deleted (by a listwise deletion strategy) due to very large missing information. Therefore, the final sample size consisted of 5003 subjects, 975 from Finland, 1866 from Poland and 2162 from Spain. The flow diagram presented in Figure 1 shows the sample selection from the 10,800 to the 5003 subject analyzed (data are available in Supplementary File 1).

### 2.2. Factors

A wide set of candidate factors (regressors) of different areas, such as socio-demographic data, anthropometric measures, vision and hearing status, health status and health habits information, presence of chronic conditions status, social networks variables and built environment variables were evaluated.

#### 2.2.1. Socio-Demographic Information

Socio-demographic information included, sex, age, country, years of educational attainment, marital status (categorized into 4 categories: “married or cohabiting”, “never married”, “separated or divorced”, and “widowed”) and location, grouped into urban or rural according to country-specific definitions.

#### 2.2.2. Anthropometric and Cognitive Measures

Height and weight were measured with the use of a stadiometer and a routinely calibrated electronic weighting scale respectively. BMI was calculated by dividing measured weight (in kilograms) by squared height (in meters), i.e., kg/m^2^. BMI was categorized as follows: underweight <18.4; normal 18.5–24.9; overweight 25.0–29.9; obese ≥30. According to the WHO (World Health Organization) standards, waist circumference (WC) was categorized into low risk (WC in the range 40–102 for males and 54–88 for females) and high risk (WC in the range 102.1–152 for males and 88.1–156 for females) [32]. In addition, measure of Handgrip (kg), walking test at four meters (seconds), verbal recall and delayed recall (as number of words), digit span forward/backward (as enter the series number in the longest series repeated without error), verbal fluency (number of animals named correctly) were also collected.

#### 2.2.3. Vision and Hearing

Vision was addressed with two questions, referred to the previous 30 days, to whom respondents reported their response on a four-step scale (no problems, mild, moderate, extreme/complete problems) and that was herein dichotomized as “yes/no”.

- Distance vision, with the question “how much difficulty did you have in seeing and recognizing an object or a person you know across the road (from a distance of about 20 m)?”
- Near vision, with the question “how much difficulty did you have in seeing and recognizing an object at arm’s length (for example, reading)?” In addition, two dichotomous indicators (yes/no) concerning to the presence of cataracts (“Cloudy or blurry vision” and “Vision problems with light”) were also reported. People were defined to have near vision problems if they responded positively to at least one of the three questions.

Hearing was also addressed with two questions referred to the previous 30 days, to whom respondents reported their response on a four-step scale (no problems, mild, moderate, extreme/complete problems) and that was herein dichotomized as “yes/no”.

- Near hearing, with the response to the question “how much difficulty did you have in: hearing someone talking on the other side of the room in a normal voice (even with your hearing aid on if you use one)?”
- Problems with conversation hearing, with the response to the question “how much difficulty did you have in: hearing what is said in a conversation between several people (even with your hearing aid on if you use one)?”

#### 2.2.4. Health State Description

Respondents were asked a series of questions on the following: own health status (self-rated health in interview day), bodily aches (or pain), difficulties (in the last 30 days) due to problems with sleep, feeling tired, work (or household activities), washing of the body, getting dressed, getting to and using the toilet, personal relationships or participation in the community, dealing with conflicts and tensions and dealing with strangers. Oral health was also recorded as dichotomous response (poor/good) to “During the last 12 months, have you had any problems with your mouth and/or teeth, including problems with swallowing?”. All items were dichotomized (yes/no in respect of problem or difficulties and poor/good for health status).

With regard to personal mobility, we developed for the purpose of this study a synthetic mobility index based on the response to the following activities: standing for long periods such as 30 min; climbing one flight of stairs without resting; vigorous activities; sitting for long periods; stooping, kneeling or crouching; picking up things with fingers; extending arms above shoulder level; walking a long distance such as a kilometer; carrying things; moving around inside home; getting up from lying down; getting where you want to go, using private or public transport if needed. Each of them was rated by respondents thinking how much of a difficulty (no, mild, moderate, severe, extreme) they had in the previous month. Items addressing difficulties in walking 100 m, standing up from sitting down, and in getting out of you home were removed for local dependence and item misfit. The one-factor mobility score ranges from 0 to 100, with higher scores indicating better mobility.

Injuries were also recorded by three dichotomous responses (yes/no) to the following questions: (a) “Did you suffer a physical disability as a result of being injured?”; (b) “In the last 12 months, have you had any other event where you suffered from bodily injury?”; (c) “In the last 12 months, have you been involved in a road traffic accident where you suffered from bodily injury?”. We defined respondents as having a physical disability as the outcome of an injury if they responded positively to one of the three questions.

Finally, both inpatient hospital and outpatient care were reported as dichotomous indicator (yes/no); concerning the latter, the health care (or consultation) times in the last 12 months were also measured. In addition, difficulty with coping was based on the response to the item “How often have you found that you could not cope with all the things that you had to do?”. Response was dichotomized as yes/no, with the “yes” category been defined by the responses “Sometimes”, “Fairly often” or “Very often”, and the “no” category by the responses “Never” or “Almost never”.

#### 2.2.5. Health Habits

Health habits included in the analysis were: smoking status, alcohol consumption, physical activity, nutrition. Concerning smoking status, respondents were classified as current smokers (yes/no) or past smokers (yes/no) vs. never smokers. With regard to alcohol consumption, questions addressed individual consumption patterns, including frequency and quantity of alcohol use. Responders were grouped into four groups [33]:

- Lifetime abstainers or occasional drinkers (i.e., those who had never consumed an alcoholic beverage or had not consumed alcohol in the last 30 days);
- Non-heavy drinkers (i.e., social drinkers who consumed alcohol in the last 30 days but were not heavy drinkers);
- Infrequent heavy drinkers (i.e., binge drinkers who consumed alcohol on 1–2 days in the past week with five or more standard drinks for men and four or more standard drinks for women);
- Frequent heavy drinkers (those who consumed alcohol on three or more days per week with five or more standard drinks for men and four or more standard drinks for women).

Questions about the type and level of physical activity that the respondent undertakes were based on the second version of the Global Physical Activity Questionnaire (GPAQ v2) [34]. The GPAQ v2 differentiates between work and leisure, and recreational and sport-related activities, and records the frequency (number of days) and duration (minutes or hours) of each activity undertaken in the preceding 7 days. Using conventional cut-off points the following levels of physical activity were created [35]:

- High physical activity (vigorous-intensity activity on at least 3 days achieving a minimum of at least 1500 Metabolic Equivalent to Task (MET)-minutes per week or seven or more days of any combination of walking, moderate or vigorous intensity activities achieving a minimum of at least 3000 MET-minutes per week);
- Moderate physical activity (3 or more days of vigorous-intensity activity of at least 20 min per day; five or more days of moderate-intensity activity or walking of at least 30 min per day; or five or more days of any combination of walking, moderate or vigorous intensity activities achieving a minimum of at least 600 MET-minutes per week);
- Low physical activity (a person not meeting any of the above mentioned criteria).

Concerning nutrition, sum of servings of the questions “How many servings of fruit do you eat on a typical day?” and “How many servings of vegetables do you eat on a typical day?” were dichotomized on <5 (yes/no) [36].

#### 2.2.6. Chronic Conditions

Presence (or absence) of chronic conditions were based on self-report by respondents through the question, “Has a health care professional ever told you, you have?” for the following eight conditions: arthritis, stroke, angina, diabetes, lung disease, asthma, depression, and hypertension.

#### 2.2.7. Social Networks

A synthetic social networks index (SNI), fully described elsewhere [37] was used to evaluate the impact of social networks. Briefly social network was defined as a multidimensional set of independent networks involving the relations with spouse, parents, other relatives (children, grandchildren and others), neighbors, friends and co-workers. For each of them, structural and functional aspects were taken into account, namely, the size of specific networks, the ties (close relations), help (general social support) and the frequency of face-to-face contacts. The SNI ranged from 0 to 100, with higher score indicating better social networks.

Interpersonal Activities were defined by dichotomous answers to three questions such: “Overall in the last 30 days, how much difficulty did you have with personal relationships or participation in the community?”; “Overall in the last 30 days, how much difficulty did you have in dealing with conflicts and tensions with others?”; “Overall in the last 30 days, how much difficulty did you have with dealing with people you do not know?”.

#### 2.2.8. Built Environment

Courage Built Environment self-reported questionnaire (CBE-SR) has been fully described elsewhere [38]. It comprises 19 items grouped into four indexes: “Usability of the neighborhood environment”, “Hindrance of walkable environment”, “Easiness of use of public buildings, places and facilities”, “Usability of the living place”. For each of the four scales, scores range between 0 and 100: higher scores address, respectively, a neighborhood environment perceived as more usable, walkable environment perceived as more hindering, public buildings, places and facilities perceived as more easy to use, and living place perceived as less risky and more usable.

### 2.3. Statistical Analyses

Descriptive statistics are expressed as mean ± standard deviation for quantitative variables and frequencies for categorical variable.

Firstly, a preliminary analysis by simple logit quasi-binomial regression models (to manage the overdispersion) was performed to evaluate the crude association (i.e., regression coefficient, *β*) of each candidate regressor on dichotomous unemployment outcome (1 = unemployed, 0 = employed). The regression coefficients were tested with *T*-test. The exponential transformation of the parameter associated with the regressor allowed a more easily interpretable odds ratio (ORs) to be obtained, on which *p*-values, and 95% confidence intervals (95% CIs) were calculated. The significant level was set at *p* = 0.05 (2-sided). Preliminary analysis was carried out also stratified by country in order to detect the possible features across crude associations.

Thus, we performed a multiple analysis by fitting multiple logit quasi-binomial regression models to evaluate the adjusted associations of the regressors with unemployment. At this stage, the associations are conditional, i.e., expected outcome variation (in OR terms, too) per unit increase of the regressor, keeping fixed the others in the built-in model. Furthermore, we carried out a model selection procedure, using forward (and backward) stepwise strategies by adding (removing) regressors to the null (full) model. In the case of the forward stepwise strategy, the algorithm included the most significant regressor and then reconsidered all included regressors for being excluded from the model. For both inclusion and deletion a *p*-value equal to 0.10 was set. On the contrary, with backward stepwise strategy, from the full model (including all regressors whose *p*-values of the simple models were less than 0.10) we removed regressors with *p*-values bigger than 0.10. The model selection procedure have been performed both on the overall sample and by country (i.e., stratified analysis) to detect differences in the adjusted associations. Multi-collinearity was checked using the generalized Variance Inflation Factor (gVIF) [39]: regressors with gVIF > 2.5 were discarded from the analysis.

In order to generate nationally representative estimates, the sample weighting and the complex study design were taken into account in all analyses (for further details see [40,41,42,43]). Hence, survey type estimations [44] were also used for the model building by considering the nature of the complex sample design, including the individual weights, cluster and strata. Accounting for this, for each multiple logit quasi-binomial regression model, design-adjusted Cox-Snell pseudo-*R*^2^ (*R*^2^) [45] was computed to evaluate the goodness of fit. Finally, design-adjusted Akaike Information Criterion (dAIC) [46] was also computed. dAIC served the scope to select the best model between two strategies, namely the forward and backward stepwise model selections: the strategy returning the lower dAIC was selected. The analysis was performed on R 3.3.2 [47] using R/survey [48,49], R/jtools [50], and R/car [39] packages.

## 3. Results

### 3.1. Characteristics of the Study Sample

The final sample with complete information across all variables comprised 5003 participants, mean age 48.11 ± 11.52) and is described in Table 1. Most of respondents were from urban contexts and the sample was slightly unbalanced for gender. Overall prevalence of unemployment was 32% and responders from Spain reported higher percentage (41%). Table 1 also reports the descriptive statistics stratified by country.

### 3.2. Regression Analysis

Table 2 and Table S1 show the results of the backward stepwise model selection, i.e., the fit results of the best selected model: the model based on the backward strategy had the lower dAIC (5170.33) and was therefore selected.

Table S1 compares crude associations with the adjusted ones, while Table 2 provides the list of the selected regressors by area. All the explanatory variables returned a gVIF less than 2.5, thereby confirming the adequacy of the model regarding its parameter estimates.

What has to be noted is that the majority of variables that were significant regressors when considered alone became not relevant when adjustment for the whole set of variables was performed. Second, for two variables, namely age and distant vision, the direction of the association changed between the simple and multiple model. In the simple models, in fact, older age and impaired distant vision were risk factors for unemployment, while in the multiple model they were protective factors.

A total of 20 variables resulted significant regressors of the multiple model (see Table 2): with the exclusion of those connected to social networks and built environment, variables from all the sections of COURAGE protocol were retained in the model. In particular, the most represented sections were those referred to health state description, anthropometric measures and cognitive functioning, and sociodemographic variables.

Factors related to the presence of employment included: some sociodemographic variables, namely higher years of education and older age and being married or separated/divorced, contrasted to being never married; anthropometric and cognitive functioning, namely handgrip strength, digit span forward and verbal fluency; problems with distant vision; moderate use of alcohol contrasted to being abstainers or occasional drinkers.

Factors related to unemployment included: sociodemographic variables, namely living in Spain, contrasted to living in Finland; anthropometric and cognitive functioning, namely high-risk WC and higher time to complete the four-meters walking test; problems with near vision and with conversation hearing; health state variables, namely inpatient care in the previous 12 months (and the number of outpatient visits), poor self-rated health status, suffering a physical disability as a result from injury, having difficulties with the use of the toilet, difficulties with personal relationship or participation in the community; diagnosis of depression.

Finally, it is worth to point out that some chronic conditions may not have been included in the selected model because masked from the self-reported health variable which might be a better regressors of health status than asking about specific conditions. By the way, significant associations between the self-reported health variable and some chronic conditions as depression (OR = 5.91, *p* < 0.001), arthritis (OR = 3.99, *p* < 0.001), Lung disease (OR = 11.27, *p* < 0.001), Hypertension (OR = 4.31, *p* < 0.001), diabetes (OR = 4.82, *p* < 0.001), angina (OR = 7.12, *p* < 0.001), stroke (OR = 7.66, *p* < 0.001) and asthma (OR = 1.73, *p* = 0.032) were observed.

### 3.3. Stratified Analysis by Country

Table 2 and Table S1 also show the results of the model selection procedures performed by country, Finland, Poland and Spain.

#### 3.3.1. Finland

The model selection provided the best model by the forward stepwise procedure (dAIC = 734.62, *R*^2^ = 0.196). Similarly to the overall model, years of education (OR = 0.92), handgrip (OR = 0.982), walking test (OR = 1.86), inpatient care (OR = 4.77) were associated to unemployment. Relevant specificities, i.e., factors just included in the Finnish selected model (irrespective from the inclusion in the overall model), generated significant associations with depression (OR = 2.46), cloudy vision (due to the cataract) (OR = 2.75), difficulty in dealing with stranger people (OR = 5.35), high physical activity (OR = 0.46) and with injuries, both from road traffic accident (OR ≈ 0) and general bodily ones (OR = 0.36).

#### 3.3.2. Poland

The model selection provided the best model by the backward stepwise procedure (dAIC = 1762.51, *R*^2^ = 0.186). Also for the Polish sample, factors as years of education (OR = 0.86), handgrip (OR = 0.964), inpatient care (OR = 1.75), self-rated health status (OR = 4.14), and a moderate alcohol consumption (OR = 0.37) were significantly associated to unemployment. Relevant specificities of this model, i.e., factors just included in the Polish selected model (irrespective from the inclusion in the overall model), were the significant relationships with the waist circumference related risk factor (OR = 1.94), conversation hearing problems (OR = 2.35), difficulties in washing the body (OR = 2.16), and the age (OR = 0.973) conversely associated.

#### 3.3.3. Spain

The model selection provided the best model by the backward stepwise procedure (dAIC = 2567.65, *R*^2^ = 0.13). Similarly to the overall model, regressors as years of education (OR = 0.94), handgrip (OR = 0.964), self-rated health status (OR = 2.27), inpatient care (OR = 1.54), and a moderate alcohol consumption (OR = 0.68) were significantly associated to unemployment. As a specificity, i.e., factors just included in the Spanish selected model (irrespective from the inclusion in the overall model), the factor relative to difficulty with personal relationships (OR = 2.72), the outpatient care (OR = 1.03), physical disability (OR = 3.07), and the digit span forward score (OR = 0.81) were significantly linked. Finally, it is worth to point out that the Polish and Spanish samples were larger, therefore their significant relationships mainly resulted in the overall selected model.

## 4. Discussion

In this study we report on selected health, health-related and epidemiological factors linked to unemployment using data referring to 5003 persons from Finland, Poland and Spain. Our results show that the most important factors related to unemployment status were health status indicators such as physical disability, self-rated health, inpatient care, and difficulties with getting to the toilet, while moderate alcohol consumption and the fact of being married, or having been married were factors related to employment status. Other factors that played a significant role included presence of depression and difficulties with near vision and conversation hearing.

In the last years, in both public health and economic framework, some studies have attempted to explain the variability of the unemployment outcome by using different sets of regressors [51,52,53]. However, to our knowledge, none have carried out model selection procedures involving simultaneously regressors from different epidemiological areas as anthropometric and cognitive markers, chronic conditions, health habits factors and many others specific indicators: the inclusion of such a wide set of regressors in our analysis constitute the most relevant strength of our study. Many of the results we achieved with this study are basically consistent with previous literature. With regard to sociodemographic features, as shown in previous studies, our results showed that older respondents were protected against unemployment compared to younger ones [23,24,25,54], and that those with higher educational attainments were less likely to be unemployed [18,19,20,54]. Also, the report from Schuring and colleagues [54] showed that exit from paid employment was more prevalent among individuals living in the Southern European region: consistently with this result, we showed that respondents living in Spain were more likely to be unemployed compared to those living in Finland. On the contrary, we did not find that females were more likely to be unemployed in the multiple model, but such a relation was found in the simple model: this provides further evidence on the importance of addressing relevant factors together, and not by addressing single relationships.

Studies addressing the impact of health on employment status generally found that people with worse health, or with chronic health conditions, also show lower employment rates [3,12,13,14,15,16,17]. Our results are generally consistent with such literature finding. Self-rated health outcomes were found in our study to be a factor pertinent for unemployment. In fact, poor self-rated health seems to be associated to unemployment condition, which is in line with previous literature showing that unemployment is associated to highest risk of hospitalization and the use of health services [55], and in general self-reported poor health was found as a significant risk factor for unemployment in a literature review [56]: this review also found similar results for poor mental health and presence of chronic conditions.

Previous literature found that people with chronic diseases have a higher risk of unemployment and inactivity as they may experience relatively quick transition paths from employment to unemployment/inactivity [57]. Our results on the impact of chronic conditions seem to point in an opposite direction: in fact, while in the simple model almost all the chronic conditions were risk factors for unemployment, only depression was retained in the full multiple model, together with the presence of physical disability as an outcome from injuries. The finding about depression as factor related to unemployment is in line with recent literature showing that the relationship between unemployment and depression is significant among adults [58,59,60]. This factor may act as a risk factor for unemployment but also as a consequence of unemployment and has been show that this population may benefit from employment and mental health focused interventions [60]. In addition, it has already been observed that the economic recession, during which our study was carried out, determined increased disparities in unemployment rates between people with and without mental health problems, and people with mental health problems are at risk of experiencing exclusion from labor market, in particular if they have low education and younger age [51]. Our results are completely in line with this finding.

Increased BMI, obesity and high-risk WC were found as regressors in the simple model, but only high-risk WC was retained—and with weak association—in the multiple model. Previous studies failed in precisely addressing the presence of some relationships between overweight, obesity and unemployment status, which seem to be connected to other factors, such as physical inactivity and smoking [61,62,63]. In addition to this, weight gain has been associated also to unemployment and job loss [63,64]. Our results do not provide univocal indications on the link of obesity and overweight on unemployment status. However, it is of interest to see that high-risk WC, which is an indicator of abdominal obesity, was associated to unemployment, similarly to what found in previous studies [65,66,67]. It has to be noted that BMI may result in important misclassification of individuals into obesity categories as it does not distinguish fat from muscle, bone and other lean body mass [68,69,70].

With regard to sensory functions, we found that people with hearing impairments, that makes it difficult to understand a conversation, were more likely to be unemployed. Difficulties with understanding conversations, clearly expose to problems with receiving information on job duties and thus respecting job schedules. It is therefore little surprising that both our results and previous literature [71,72] found an association between hearing difficulties and unemployment. Parallel to this, we found that unemployment was predicted by low near vision capacity, whose presence clearly has an impact on the majority of work-related tasks, i.e., those based on offices, stores or factories, as also shown in previous studies [73,74], and we believe that this is the path leading to unemployment in this group.

With regard to health habits and risk factors for health, our data show that in simple models current smoking was a risk factor for unemployment, while moderate alcohol consumption and high physical activity levels were protective factors: however, in the multiple model, only moderate alcohol consumption was retained. Moderate alcohol consumption is a known protective factor for health [75], while evidence exists that risky alcohol consumption (associated with hazardous, binge, and heavy drinking) is more prevalent among the unemployed [76]. It is therefore possible to suppose that the protective effect of moderate alcohol consumption is also connected to the general protective effect of good health outcomes that was found in previous literature as well as in our study.

Our results can be broadly divided in two areas: the factors related to unemployment that are not, or little, likely to be modified if not in the long run, and those that might be modified with short or medium term interventions. Among this group, variables such as handgrip strength, alcohol consumption, depression, WC, which is a cardiovascular risk factor, and 4-m walking speed are included. Some of these outcomes, i.e., handgrip strength, WC and 4-m walking speed, may be targeted by increasing the level of people physical activity [77,78,79]: increasing physical activity directly enhances people’s health, which in turn is expected to increase employment of populations [3,11]. In particular, handgrip is a measure of physical fitness that is quite easy to assess in an outpatient practice. Therefore, since most unemployed people would have to arrange their own programs to increase their fitness, measuring hand grip could be a way to objectify their progress. The benefit of moderate alcohol consumption on health have been widely addressed, as shown in a systematic literature review [75]. Current moderate drinkers have been shown to self-report and objectively be in a better health compared to and heavy drinkers [80,81,82], but confounding may occur on the inclusion (or exclusion) of abstainers that might have decreased their level of alcohol consumption due to ill health or due to problems with alcohol [83]. Research has also shown a negative association between heavy drinking and employment status [84,85,86], and a dose-effect relationship between alcohol consumption and early work cessation due to death, disability and early retirement, with retirement before the age of 55 being the most common cause [87]. Concerning stratified analysis, previous studies have confirmed that people with lower level of education that were working in low-skilled jobs before economic recession were the more frequently unemployed during the harder recession years in Spain and Finland [88,89]. It is also in line with literature that general and physical health status and unemployed are interrelated, well because unemployment cause a decrement in health status [90], well because people with ill-health have more difficulties to access employment [91] or more likely to become unemployed [92,93,94]. In particular, previous studies highlighted specific associations with depression in Finland [95,96] and with hospital services use [55] and shock-related physical disabilities [97,98] in Spain. Also in Poland, although it has not experienced the economic recession, lower level of education and poor health status were linked with unemployment. Particularly, a previous study on a Polish sample stated a significant association between cardiovascular risk factors and unemployment in men [99].

In this country, it is also worth to highlight as different factors regarding visual problems were associated with unemployment, especially by simple models. In Finland and Spain, only specific problems due to cataracts were related. In general, these results follow the evidence yielded from surveys in Europe [100] and in the World [74], that states as the visual difficulty is associated to (un)employment status.

It is worth mentioning the link with difficulties in personal relationships and in dealing with strangers with unemployment in Spain and Finland, respectively. Again several interpretations are possible. One of them is related to the fact unemployed people might have lower social contacts than employed (due to the coworkers contacts) [101]. Finally, another interpretation could be related to the fact people with lower social support have lower job search efficacy and therefore they might be more likely unemployed [102].

The strengths of this study lie in the wide sample size, and in the fact that it was drawn from three different European countries, as well as in its relying on a comprehensive set of clinical and epidemiological factors related to unemployment that in most of previous literature have been individually addressed, while in this study were included in the multiple models. In particular, beyond the evaluation of the crude associations, model selection procedures have provided estimates of adjusted associations and a focused regressors set, overall and by stratifying by country (i.e., Finland, Poland and Spain). For this reason, causal relationships might be hypothesized to address theoretical dynamics of confounding, mediation or moderation among the selected regressors disclose by this study. The result of this was the possibility to provide indications that, first of all, may be valid for a Europe-wide context, as differences in socio-economic gradients between Poland and Spain and Finland exist. For example, Spain is a country that is ageing very rapidly, with very low institutionalization rates, still represents a culture in which families play a key role in taking care of individuals with employment problems, and has also experienced great demographic changes, with a flow of immigrants which has increased the population and had an impact on the workforce. Conversely, Poland is the largest of the newer member states and has a very rapidly ageing population, comparable to most of Western Europe. Finally, Finland is the richest of the three countries and that with the smallest population, with a strong social welfare system. The second relevant strength lies in the possibility, given by the inclusion of a large amount of variables, to delineate indications for actions that can be carried out at short-medium term, thus with the possibility to transfer the result of the survey into concrete actions whose effects could be appreciated in less than a decade.

Some limitations have however to be acknowledged. First, the cross-sectional study design does not allow to identify the causal relationships, i.e., the causal mechanisms: between many of the associations observed, we do not know whether the factor associated with unemployment led to unemployment or was a consequence of becoming unemployed. For example, unemployment has effects on changes in alcohol consumption, marital relationships and leading to depression. The reality is that both associations and causation act simultaneously also in studies on unemployed cohorts [92] and the causal mechanisms depend in strong way from the specific situations. Particular, in our study we found that depression is a risk factor for unemployment, but the opposite relation cannot be excluded, as shown in previous studies [103,104]. Second, the study involves only clinical and epidemiological factors and misses to consider the probably most important one, the economic situation in the country. Moreover, we did not addressed those that were discouraged with regard to the possibility of being employed, such as the so-called NEETs (i.e., young people that are not engaged in employment, education, or training) that are at risk of long-term economic disadvantage and social exclusion, depression, functional disability and engagement in criminality [105,106]. Similarly, we did not differentiate between those who had lost their job from those who never engaged in any job or from those who were “temporarily” out of the labor market, i.e., those that are actively looking for a job, and that are likely to find one. In addition, these aspects might also have contributed to overestimate the unemployed rates, although the ranking and differences between the countries were proportionally coherent. In fact, in a period covered by the survey (i.e., December 2011), the national unemployment rates (15–74 years old) were 7.6% in Finland, 9.9% in Poland and 22.9% in Spain [107], compared to 18.15%, 29.09% and 41.16% of the COURAGE data. Accounting for this and the specificity of the study, it is worth to point out that some findings, especially in the stratified analysis, might also be due to chance or lack of power (e.g., injuries-related indicators in Finland).

Anyway, the modern labor market in Europe does not guarantee at all a long-term employment status, particularly for younger individuals, thus determining relevant inequalities within societies [108,109] and some periods of instability due to insecure or temporary employment are to be foreseen, which will in turn lead to negative social consequences. In fact, temporary employment and its association with worst working conditions and intrinsic insecurity, contributes to the creation of problems at family and private life levels, in terms of having relatively less spare time for family, experiencing higher level of conflict with partners, lower likelihood to have children, and a generally lower life satisfaction, well-being and a worse household income [110]. Finally, our definition of unemployment also included house workers, which are generally not considered in this kind of study as they do not match the strict definition of unemployment of the International Labour Organization [10]. It has however to be noted that some studies, addressing health-related QoL in samples of female workers and housewife indicate that the latter have poorer scores [110,111,112]: so the inclusion of these persons among the unemployed group is justified by the risk they have of being in a worse health. Third, health conditions were self-reported, which could lead to identification bias. To reduce the risk of reporting biases, generic reference to broad illness group was used, and therefore no definition of disease severity could be applied. Fourth, data on health state description and on difficulties in daily activities were referred to the previous thirty days, but no indication on the onset of the problem is included, and the same applies to health conditions: the result of this is that it is not possible to address whether the onset of these problems was precedent or subsequent to the unemployment status. For all of the above reasons, a cautious interpretation of the result of our study is suggested.

## 5. Conclusions

We reported information on health and epidemiological factors related to unemployment status from a wide sample of citizens from Finland, Poland and Spain. Our results show that some of these factors can be addressed with short-medium term interventions that focus on health promotion and health prevention strategies: for example, interventions aimed to promote physical activity and healthy behaviors, tackling depression or promoting education, in particular for the younger.

Data are needed for better policies able to answer to the need for better knowledge on innovative strategies to improve participation in the labor market and to the need of having a stronger focus on reintegration or inclusion into employment of all citizens. The labor market is undergoing profound changes and societies will more and more be in need of adequate information on which determinants of employment could be addressed to enhance employment rates of European citizens.

## Figures and Tables

**Table 1:** Descriptive statistics (overall and by country).

Variable (Regressor)	Total Sample N = 5003	Finland N = 975 (19.48%)	Poland N = 1866 (37.29%)	Spain N = 2162 (43.21%)
	N (%) or Mean ± SD	N (%) or Mean ± SD	N (%) or Mean ± SD	N (%) or Mean ± SD
Outcome				
Unemployment—Yes	1610 (32.18)	177 (18.15)	543 (29.09)	890 (41.16)
Socio-demographic information				
Sex—Male	2271 (45.39)	431 (44.20)	810 (43.40)	1030 (47.64)
Age (year)	48.11 ± 11.53	48.50 ± 11.23	46.61 ± 12.15	49.22 ± 10.94
Marital status				
Never married and no cohabiting	974 (19.46)	179 (18.35)	389 (20.84)	406 (18.77)
Currently married or cohabiting	3290 (65.76)	680 (69.74)	1178 (63.12)	1432 (66.23)
Separated or divorced	510 (10.19)	98 (10.05)	189 (10.12)	223 (10.31)
Widowed	229 (4.57)	18 (1.84)	110 (5.89)	101 (4.67)
Years of education completed (year)	13.14 ± 4.48	13.93 ± 3.74	13.066 ± 3.38	12.837 ± 5.46
Location—Urban	3689 (73.73)	760 (77.94)	1081 (57.93)	1848 (85.47)
Anthropometric and cognitive measures				
Height (m)	1.68 ± 0.09	1.70 ± 0.09	1.68 ± 0.09	1.65 ± 0.09
Weight (kg)	75.41 ± 15.74	77.57 ±16.12	75.14 ± 16.01	74.68 ± 15.24
Body Mass Index (BMI) (kg/m^2^)	26.84 ± 4.94	26.65 ± 4.73	26.53 ± 5.10	27.19 ± 4.87
BMI in class				
underweight	85 (1.69)	7 (0.71)	45 (2.41)	33 (1.52)
normal weight	1833 (36.63)	386 (39.58)	747 (40.03)	700 (32.37)
overweight	1958 (39.13)	390 (40)	661 (35.42)	907 (41.95)
obese	1127 (22.52)	192 (19.69)	413 (22.13)	522 (24.14)
Waist Circumference (WC) (cm)	90.43 ± 13.83	90.80 ± 13.58	88.73 ± 13.91	91.73 ± 13.71
WC cardiovascular risk factor—High	1764 (35.25)	339 (34.76)	578 (30.97)	847 (39.17)
Walking test at 4 m (seconds)	3.19 ± 1.50	2.40 ± 0.86	3.44 ± 1.93	3.34 ± 1.15
Handgrip (Kg)	36.60 ± 12.23	39.56 ± 12.90	36.73 ± 12.12	35.15 ± 11.76
Verbal recall (Number of words)	19.11 ± 5.35	22.64 ± 3.71	18.59 ± 5.57	17.96 ± 5.11
Delayed verbal recall (Number of words)	6.20 ± 2.32	7.97 ± 1.66	5.89 ± 2.35	5.66 ± 2.15
Digit span forward (Enter the series number in the longest series repeated without error)	5.88 ± 1.44	6.07 ± 1.12	5.66 ± 1.59	5.98 ± 1.42
Digit span backward (Enter the series number in the longest series repeated without error)	4.16 ± 1.40	4.63 ± 1.24	3.98 ± 1.58	4.09 ± 1.25
Verbal fluency (Number of animals named correctly)	21.76 ± 8.35	26.18 ± 7.29	21.27 ± 8.34	20.18 ± 8.12
Vision and Hearing				
Distance Vision—Poor	855 (17.08)	38 (3.89)	342 (18.32)	475 (21.97)
Near Vision—Poor	650 (12.99)	10 (1.02)	574 (30.76)	66 (3.05)
Cloudy or blurry vision due to cataracts—Yes	375 (7.49)	61 (6.25)	174 (9.32)	140 (6.47)
Vision problems with light due to cataracts—Yes	357 (7.13)	77 (7.89)	158 (8.46)	122 (5.64)
Near Hearing—Poor	519 (10.37)	132 (13.53)	200 (10.71)	187 (8.64)
Conversation Hearing—Poor	432 (8.63)	59 (6.05)	235 (12.59)	138 (6.38)
Health State				
Health status (self-rated health status interview day)—Poor	424 (8.47)	34 (3.48)	178 (9.53)	212 (9.80)
Difficult with work or household activities (since 30 days)—Yes	1427 (28.52)	182 (18.66)	698 (37.40)	547 (25.30)
Difficulties in coping—Yes	1653 (33.04)	108 (11.07)	795 (42.60)	750 (34.69)
Bodily aches or pains—Yes	2676 (53.48)	662 (67.89)	1056 (56.59)	958 (44.31)
Mobility (score)	91.45 ± 16.07	94.81 ± 11.27	88.45 ± 18.45	92.51 ± 15.26
Difficulty in washing the whole body task—Yes	305 (6.09)	38 (3.89)	175 (9.37)	92 (4.25)
Difficulty in getting dress—Yes	367 (7.33)	41 (4.20)	205 (10.98)	121 (5.59)
Difficulty with getting to and using the toilet?—Yes	203 (4.05)	15 (1.53)	132 (7.07)	56 (2.59)
Difficulty with personal relationships or participation in the community?—Yes	449 (8.97)	78 (8)	245 (13.12)	126 (5.82)
Difficulty in dealing with conflicts and tensions with others?—Yes	645 (12.89)	142 (14.56)	352 (18.86)	151 (6.98)
Difficulty with dealing with people you do not know?—Yes	457 (9.13)	73 (7.48)	263 (14.09)	121 (5.59)
Difficulty in sleep—Yes	1917 (38.31)	444 (45.53)	749 (40.13)	724 (33.48)
Feel tired—Yes	1948 (38.93)	543 (55.69)	777 (41.63)	628 (29.04)
Oral health—Poor	1017 (20.32)	327 (33.53)	217 (11.62)	473 (21.87)
Road Traffic Accident Injuries -Yes	95 (1.90)	10 (1.02)	35 (1.87)	50 (2.31)
General Bodily Injuries—Yes	272 (5.43)	87 (8.92)	75 (4.01)	110 (5.08)
Physical Disability (from injury)—Yes	59 (1.18)	9 (0.92)	16 (0.85)	34 (1.57)
Inpatient care—Yes	1011 (20.20)	192 (19.69)	441 (23.63)	378 (17.48)
Outpatient care—Yes	2638 (52.72)	727 (74.56)	518 (27.75)	1393 (64.43)
Outpatient care (times in the last 12 months)	2.67 ± 6.64	3.92 ± 10.00	1.70 ± 4.88	2.95 ± 5.89
Health Habits				
Current smoking status—Yes	1697 (33.91)	223 (22.87)	679 (36.38)	795 (36.77)
Past smoking status—Yes	989 (19.76)	315 (32.30)	344 (18.43)	330 (15.26)
Alcohol consumption				
Abstainer or occasional	2726 (54.48)	385 (39.48)	1158 (62.05)	1183 (54.71)
Drinker/Not Heavy Drinker	1786 (35.69)	346 (35.48)	550 (29.47)	890 (41.16)
Infrequent Heavy Drinker	437 (8.73)	223 (22.87)	141 (7.55)	73 (3.37)
Frequent Heavy Drinker	54 (1.07)	21 (2.15)	17 (0.91)	16 (0.74)
Physical activity				
Inactive or low	941 (18.80)	112 (11.48)	353 (18.91)	476 (22.01)
Moderate	1595 (31.88)	376 (38.56)	370 (19.82)	849 (39.26)
High	2467 (49.31)	487 (49.94)	1143 (61.25)	837 (38.71)
Fruit or vegetable nutrition (servings per day <5)—Yes	3825 (76.45)	775 (79.48)	1455 (77.97)	1595 (73.77)
Chronic Conditions				
Arthritis	915 (18.28)	287 (29.43)	318 (17.04)	310 (14.33)
Stroke	65 (1.29)	9 (0.92)	33 (1.76)	23 (1.06)
Angina	164 (3.27)	13 (1.33)	97 (5.19)	54 (2.49)
Diabetes	333 (6.65)	55 (5.64)	118 (6.32)	160 (7.40)
Lung disease	204 (4.07)	17 (1.74)	96 (5.14)	91 (4.20)
Asthma	324 (6.47)	106 (10.87)	90 (4.82)	128 (5.92)
Depression	756 (15.11)	145 (14.87)	192 (10.28)	419 (19.38)
Hypertension	1094 (21.86)	185 (18.97)	491 (26.31)	418 (19.33)
Social Network				
Social Network index (score)	69.45 ± 13.11	64.79 ± 10.74	66.03 ± 12.85	74.48 ± 12.57
Build Environment Assessment scores				
Reachability and usability of the neighborhood environment	64.95 ± 21.86	58.25 ± 17.90	62.79 ± 23.87	69.82 ± 20.54
Hindrance of walkable environment	27.93 ± 19.96	20.14 ± 10.50	34.40 ± 22.77	25.85 ± 18.90
Open-to-public buildings, places and facilities	72.88 ± 19.84	75.69 ± 12.06	63.81 ± 22.40	79.44 ± 17.17
Usability of the living place/home	78.13 ± 19.37	83.07 ± 11.93	70.65 ± 22.72	82.34 ± 16.80

**Table 2:** Regressors of the selected multiple models divided by area.

Areas	Regressors	Adjusted OR (95% CI)			
		Overall N = 5003	Finland N = 975	Poland N = 1866	Spain N = 2162
Socio-demographic information	Years of education completed	0.92 *** [0.90; 0.95]	0.92 * [0.86; 0.98]	0.86 *** [0.82; 0.91]	0.94 *** [0.91; 0.97]
	Country (ref. Finland)		Not expected	Not expected	Not expected
	Poland	1.01 [0.74; 1.40]	Not expected	Not expected	Not expected
	Spain	2.17 *** [1.67; 2.83]	Not expected	Not expected	Not expected
	Marital status (Ref. never married)		Not included	Not included	Not included
	Currently married or cohabiting	0.73 * [0.56; 0.96]			
	Separated or divorced	0.58 ** [0.39; 0.87]			
	Widowed	0.92 [0.52; 1.61]			
	Age (years)	0.991 * [0.982; 0.999]	Not included	0.973 *** [0.95; 0.98]	Not included
Anthropometric and cognitive measures	Handgrip	0.979 *** [0.969; 0.988]	0.982 * [0.967; 0.998]	0.964 *** [0.948; 0.981]	0.983 * [0.971; 0.996]
	Digit span forward	0.89 ** [0.82; 0.97]	Not included	Not included	0.81 *** [0.72; 0.91]
	WC cardiovascular risk factor (ref. Low)	1.26 ° [0.99; 1.59]	Not included	1.94 ** [1.24; 3.02]	Not included
	Walking test at 4 m (seconds)	1.08 ° [0.99; 1.17]	1.86 ** [1.20; 2.88]	1.12 ° [1.00; 1.28]	Not included
	Verbal fluency	0.99 ° [0.97; 1.00]	Not included	Not included	Not included
Vision and hearing	Distant Vision (ref. Good)	0.69 ** [0.53; 0.89]	Not included	Not included	Not included
	Near Vision (ref. Good)	1.58 * [1.10; 2.28]	Not included	1.48 ° [1.00; 2.19]	Not included
	Cloudy or blurry vision due to cataracts (ref. No)	Not included	2.75 * [1.17; 6.47]	Not included	Not included
	Conversation Hearing (ref. Good)	1.60 * [1.10; 2.31]	Not included	2.35 ** [1.38; 3.98]	Not included
Health state	Inpatient care (ref. No)	1.98 *** [1.54; 2.55]	4.77 *** [2.94; 7.75]	1.75 * [1.12; 2.70]	1.54 * [1.06; 2.22]
	Self-rated health status (ref. Good)	2.63 *** [1.75; 3.98]	Not included	4.14 *** [2.20; 8.04]	2.27 ** [1.29; 4.07]
	Physical Disability (from injury) (ref. No)	2.94 ** [1.47; 6.04]	Not included	Not included	3.07 * [1.21; 8.51]
	Difficulty with getting to and using the toilet? (ref. No)	2.04 ** [1.26; 3.33]	Not included	Not included	Not included
	Difficulty in washing the whole body task (ref. No)	Not included	Not included	2.16 ** [1.23; 3.79]	Not included
	Difficulty with personal relationships or participation in the community? (ref. No)	1.45 ° [0.96; 2.18]	Not included	Not included	2.72 ** [1.45; 5.23]
	Difficulty in dealing with conflicts and tensions with others (ref. No)	Not included	Not included	Not included	0.53 ° [0.28; 1.01]
	Difficulty with dealing with people you do not know (ref. No)	Not included	5.35 *** [2.61; 10.99]	Not included	Not included
	Outpatient care (times in the last 12 months)	1.03 ° [1.00; 1.06]	Not included	Not included	1.03 * [1.01; 1.07]
	Road Traffic Accident Injuries (ref. No)	Not included	≈0 *** [≈0; ≈0]	Not included	Not included
	General Bodily Injuries (ref. No)	Not included	0.36 * [0.15; 0.84]	Not included	Not included
Health Habits	Alcohol consumption (ref. Abstainer/Occasional)		Not included		
	Non-Heavy Drinker	0.73 ** [0.59; 0.90]		0.77 [0.52; 1.13]	0.68 ** [0.52; 0.89]
	Infrequent Heavy Drinker	0.57 ** [0.40; 0.81]		0.37 ** [0.18; 0.73]	0.74 [0.36; 1.47]
	Frequent Heavy Drinker	1.12 [0.53; 2.25]		0.83 [0.22; 2.46]	0.64 [0.13; 2.60]
	Physical activity (ref. Inactive or low)	Not included		Not included	Not included
	Moderate physical activity		0.50 ° [0.25; 1.00]		
	High physical activity		0.46 * [0.24; 0.92]		
Chronic Conditions	Depression (ref. No)	1.38 * [1.03; 1.85]	2.46 ** [1.36; 4.46]	Not included	Not included
	Arthritis (ref. No)	Not included	Not included	Not included	1.46 ° [0.97; 2.18]
	Asthma (ref. No)	Not included	Not included	0.49 ° [0.22; 0.97]	Not included
Build Environment Assessment	Reachability and usability of the neighborhood environment	Not included	Not included	0.993 ° [0.98; 1.00]	Not included
	Usability of the living place/home	Not included	Not included	Not included	0.992 ° [0.984; 1.001]

OR = Odds ratio, 95% CI = 95% Confidence Interval, *p* = *p*-value. *** = *p* < 0.001, ** = 0.01 < *p* < 0.001, * = 0.05 < *p* < 0.01, ° = 0.05 < *p* < 0.10. ref.: reference.

## Supplementary Materials

The following are available online at http://www.mdpi.com/1660-4601/15/4/722/s1, Supplementary File 1: dataset used for the manuscript analyses. Table S1: Results of the simple and selected multiple logit quasi-binomial regression models overall and by country: crude and adjusted associations (i.e., odds ratio) with unemployment.
