# Supplementary material for: Factors Related to Unemployment in Europe. A Cross-Sectional Study from the COURAGE Survey in Finland, Poland and Spain
Source: Int J Environ Res Public Health. 2018 Apr 11;15(4):722. doi: 10.3390/ijerph15040722 (PMC5923764; doi:10.3390/ijerph15040722)
Supplement: Supplementary file 1 [file ijerph-15-00722-s001.zip › ijerph-291738-suppl/ijerph-281738-suppl.docx]

***Supplementary material***

Factors Related to Unemployment in Europe.
A Cross-Sectional Study from the COURAGE Survey in Finland, Poland and Spain

**Table S1.** Results of the simple and selected multiple logit quasi-binomial regression models overall and by country: crude and adjusted associations (i.e., dds ratio) with unemployment.

| **Regressors** | **Simple models** | | | | **Multiple models** | | | |
| --- | --- | --- | --- | --- | --- | --- | --- | --- |
|  | Overall  N = 5003 | Finland  N = 975 | Poland  N = 1866 | Spain  N = 2162 | Overall  (by *backward stepwise* model selection procedure)  *DCS pseudo-R^2^ = 0.166*  *dAIC = 5170.33* | Finland  (by *forward stepwise* model selection procedure)  *DCS pseudo-R^2^ = 0.196*  *dAIC = 734.62* | Poland  (by *backward stepwise* model selection procedure)  *DCS pseudo-R^2^ = 0.186*  *dAIC = 1762.51* | Spain  (by *backward stepwise* model selection procedure)  *DCS pseudo-R^2^ = 0.130*  *dAIC = 2567.65* |
|  | Crude OR  [95%CI] | | | | Adjusted OR  [95%CI] | | | |
| **Socio-demographic Information** | | | | | | | | |
| Sex (ref. Female) | 0.60 ***  [0.50; 0.73] | 0.56 ***  [0.42; 0.76] | 0.51 ***  [0.35; 0.76] | 0.64 **  [0.49; 0.84] | Not included | Not included | Not included | Not included |
| Age | 1.011 **  [1.004; 1.019] | 1.005  [0.988; 1.023] | 1.015 *  [1.002; 1.028] | 1.01 *  [1.00; 1.02] | 0.991 *  [0.982; 0.999] | Not included | 0.973 ***  [0.95; 0.98] | Not included |

**Table S1.** *Cont.*

| Marital status (ref. Never married / no cohabiting) |  |  |  |  |  | Not included | Not included | Not included |
| --- | --- | --- | --- | --- | --- | --- | --- | --- |
| Currently married or cohabiting | 0.81 °  [0.65; 1.02] | 0.68  [0.42; 1.11] | 0.86  [0.52; 1.45] | 0.88  [0.66; 1.19] | 0.73 *  [0.56; 0.96] |  |  |  |
| Separated or divorced | 0.95  [0.65; 1.36] | 0.99  [0.43; 2.11] | 0.99  [0.52; 1.83] | 0.90  [0.52; 1.54] | 0.58 **  [0.39; 0.87] |  |  |  |
| Widowed | 1.68 °  [0.98; 2.86] | 1.33  [0.32; 4.42] | 1.36  [0.48; 3.59] | 2.85 ***  [1.68; 4.91] | 0.92  [0.52; 1.61] |  |  |  |
| Years of education completed | 0.90 ***  [0.88; 0.92] | 0.89 ***  [0.85; 0.94] | 0.86 ***  [0.81; 0.90] | 0.92 ***  [0.89; 0.95] | 0.92 ***  [0.90; 0.95] | 0.92 *  [0.86; 0.98] | 0.86 ***  [0.82; 0.91] | 0.94 ***  [0.91; 0.97] |
| Location (ref. urban) | 1.02  [0.81; 1.29] | 1.09  [0.67; 1.81] | 1.35  [0.91; 2.00] | 0.93  [0.68; 1.26] | Not included | Not included | Not included | Not included |
| Country (ref. Finland) |  | Not expected | Not expected | Not expected |  | Not expected | Not expected | Not expected |
| Poland | 1.48 **  [1.16; 1.90] |  |  |  | 1.01  [0.74; 1.40] |  |  |  |
| Spain | 2.53 ***  [2.06; 3.12] |  |  |  | 2.17 ***  [1.67; 2.83] |  |  |  |
| **Anthropometric and Cognitive Measures** | | | | | | | | |
| BMI | 1.04 ***  [1.02; 1.06] | 1.05 *  [1.01; 1.10] | 1.02  [0.99; 1.06] | 1.04 **  [1.01; 1.08] | Not included | Not included | Not included | Not included |
| BMI in class (ref. Normal) |  |  |  |  | Not included | Not included | Not included | Not included |
| Underweight | 1.42  [0.78; 2.52] | 2.54  [0.73; 7.64] | 1.23  [0.48; 2.87] | 1.36  [0.49; 3.58] |  |  |  |  |
| Overweight | 1.13  [0.91; 1.40] | 1.24  [0.86; 1.76] | 1.04  [0.67; 1.58] | 1.12  [0.83; 1.52] |  |  |  |  |
| Obese | 1.55 ***  [1.20; 2.01] | 1.86 *  [1.12; 3.06] | 1.17  [0.72; 1.87] | 1.72 **  [1.21; 2.46] |  |  |  |  |
| WC cardiovascular risk factor (ref. Low) | 1.63 ***  [1.34; 1.98] | 1.79 **  [1.18; 2.73] | 2.03 ***  [1.37; 3.03] | 1.30 *  [1.01; 1.69] | 1.26 °  [1.00; 1.59] | Not included | 1.94 **  [1.24; 3.02] | Not included |
| Walking test at 4 meters (seconds) | 1.25 **  [1.09; 1.46] | 3.57 ***  [2.46; 5.18] | 1.23 *  [1.04; 1.50] | 1.09  [0.93; 1.29] | 1.08 °  [1.00; 1.17] | 1.86 **  [1.20; 2.88] | 1.12 °  [1.00; 1.28] | Not included |
| Handgrip | 0.968 ***  [0.960; 0.977] | 0.972 ***  [0.960; 0.985] | 0.962 ***  [0.944; 0.981] | 0.976 ***  [0.965; 0.987] | 0.979 ***  [0.969; 0.988] | 0.982 *  [0.967; 0.998] | 0.964 ***  [0.948; 0.981] | 0.983 *  [0.971; 0.996] |
| Verbal recall | 0.94 ***  [0.92; 0.96] | 0.93 *  [0.88; 0.98] | 0.97  [0.94; 1.01] | 0.93 ***  [0.91; 0.96] | Not included | Not included | Not included | Not included |
| Delayed verbal recall | 0.88 ***  [0.85; 0.92] | 0.89 °  [0.79; 1.01] | 0.94  [0.88; 1.01] | 0.87 ***  [0.82; 0.93] | Not included | Not included | Not included | Not included |
| Digit span forward (Enter the series number in the longest series repeated without error) | 0.80 ***  [0.74; 0.87] | 0.72 **  [0.60; .89] | 0.87 °  [0.75; 1.01] | 0.73 ***  [0.66; 0.81] | 0.89 **  [0.82; 0.97] | Not included | Not included | 0.81 ***  [0.72; 0.91] |
| Digit span backward (Enter the series number in the longest series repeated without error) | 0.77 ***  [0.72; 0.83] | 0.81 *  [0.70; 0.95] | 0.81 ***  [0.73; 0.91] | 0.73 ***  [0.65; 0.82] | Not included | Not included | Not included | Not included |
| Verbal fluency | 0.96 ***  [0.95; 0.97] | 0.96 **  [0.94; 0.99] | 0.96 ***  [0.93; 0.98] | 0.96 **  [0.95; 0.99] | 0.99 °  [0.97; 1.00] | Not included | Not included | Not included |
| **Vision and Hearing** | | | | | | | | |
| Distant Vision (ref. Good) | 1.26 °  [0.99; 1.60] | 1.01  [0.46; 2.27] | 1.83 **  [1.18; 2.81] | 0.81  [0.60; 1.11] | 0.69 **  [0.53; 0.89] | Not included | Not included | Not included |
| Near Vision (ref. Good) | 1.68 ***  [1.26; 2.23] | 2.98  [0.65; 13.64] | 2.32 ***  [1.60; 3.35] | 1.48  [0.63; 3.39] | 1.58 *  [1.10; 2.28] | Not included | 1.48 °  [1.00; 2.19] | Not included |
| Cloudy or blurry vision due to cataracts (ref. No) | 2.23 ***  [1.62; 3.05] | 2.92 **  [1.47; 5.80] | 2.25 ***  [1.39; 3.59] | 2.35 **  [1.29; 4.36] | Not included | 2.75 *  [1.17; 6.47] | Not included | Not included |
| Vision problems with light due to cataracts (ref. No) | 1.39 *  [1.00; 1.91] | 1.82 *  [1.09; 3.06] | 1.24  [0.72; 2.08] | 2.06 *  [1.17; 3.67] | Not included | Not included | Not included | Not included |
| Near Hearing (ref. Good) | 1.48 **  [1.10; 1.99] | 0.97  [0.58; 1.64] | 1.94 *  [1.10; 3.36] | 1.88 *  [1.16; 3.05] | Not included | Not included | Not included | Not included |
| Conversation Hearing (ref. Good) | 2.14 ***  [1.51; 3.00] | 1.98 °  [0.99; 3.96] | 2.34 **  [1.39; 3.91] | 2.48 **  [1.38; 4.54] | 1.60 *  [1.10; 2.31] | Not included | 2.35 **  [1.38; 3.98] | Not included |
| **Health State** | | | | | | | | |
| Self-rated health status (ref. Good) | 6.47 ***  [4.58; 9.27] | 9.76 ***  [4.05; 23.49] | 9.05 ***  [5.51; 15.36] | 4.33 ***  [2.55; 7.62] | 2.63 ***  [1.75; 3.98] | Not included | 4.14 ***  [2.20; 8.04] | 2.27 **  [1.29; 4.07] |
| Difficult with work or household activities (since 30 days) (ref. No) | 2.11 ***  [1.73; 2.56] | 2.56 ***  [1.79; 3.68] | 2.45 ***  [1.70; 3.53] | 2.03 ***  [1.51; 2.74] | Not included | Not included | Not included | Not included |
| Difficulties in coping (ref. No) | 1.62 ***  [1.33; 1.97] | 3.37 ***  [2.01; 5.65] | 1.48 *  [1.04; 2.12] | 1.42 *  [1.06; 1.89] | Not included | Not included | Not included | Not included |
| Bodily aches or pains (ref. No) | 1.40 ***  [1.16; 1.69] | 1.59 *  [1.01; 2.51] | 2.12 ***  [1.44; 3.15] | 1.47 **  [1.14; 1.90] | Not included | Not included | Not included | Not included |
| Mobility | 0.967 ***  [0.962; 0.973] | 0.947 ***  [0.929; 0.966] | 0.964 ***  [0.955; 0.972] | 0.97 ***  [0.960; 0.981] | Not included | Not included | Not included | Not included |
| Difficulty in washing the whole body task (ref. No) | 4.61 ***  [3.11; 6.90] | 5.91 ***  [2.34; 14.92] | 4.96 ***  [2.92; 8.55] | 6.12 ***  [2.79; 15.12] | Not included | Not included | 2.16 **  [1.23; 3.79] | Not included |
| Difficulty in getting dress (ref. No) | 3.05 ***  [2.22; 4.21] | 3.44 **  [1.43; 8.25] | 3.66 ***  [2.33; 5.74] | 3.03 ***  [1.72; 5.46] | Not included | Not included | Not included | 0.548 ° |
| Difficulty with getting to and using the toilet?  (ref. No) | 5.27 ***  [3.28; 8.69] | 8.66 *  [1.40; 53.68] | 5.11 ***  [2.84; 9.39] | 8.77 ***  [4.24; 20.66] | 2.04 **  [1.26; 3.33] | Not included | Not included | Not included |
| Difficulty with personal relationships or participation in the community? (ref. No) | 2.29 ***  [1.64; 3.20] | 2.24 **  [1.26; 3.98] | 2.46 **  [1.38; 4.34] | 4.02 ***  [2.49; 6.67] | 1.45 °  [0.96; 2.18] | Not included | Not included | 2.72 **  [1.45; 5.23] |
| Difficulty in dealing with conflicts and tensions with others? (ref. No) | 1.52 **  [1.14; 2.01] | 2.31 ***  [1.44; 3.72] | 1.83 *  [1.12; 2.93] | 1.67 *  [1.06; 2.63] | Not included | Not included | Not included | 0.53 °  [0.28; 1.01] |
| Difficulty with dealing with people you do not know? (ref. No) | 1.98 ***  [1.51; 2.60] | 5.48 ***  [3.04; 9.88] | 1.57 *  [1.03; 2.35] | 2.77 ***  [1.66; 4.72] | Not included | 5.35 ***  [2.61; 10.99] | Not included | Not included |
| Difficulty in sleep (ref. No) | 1.52 ***  [1.27; 1.83] | 1.77 **  [1.25; 2.51] | 1.66**  [1.13; 2.43] | 1.76 ***  [1.36; 2.28] | Not included | Not included | Not included | Not included |
| Feel tired (ref. No) | 1.20 °  [0.99; 1.43] | 2.13 **  [1.33; 3.41] | 1.23  [0.85; 1.78] | 1.55 **  [1.198; 2.029] | Not included | Not included | Not included | Not included |
| Oral health (ref. Good) | 1.12  [0.91; 1.37] | 1.46 *  [1.09; 1.97] | 1.01  [0.55; 1.78] | 1.15  [0.86; 1.54] | Not included | Not included | Not included | Not included |
| Road Traffic Accident Injuries (ref. No) | 0.79  [0.40; 1.44] | ≈0 ***  [≈0; ≈0] | 0.35  [0.08; 1.05] | 1.04  [0.44; 2.30] | Not included | ≅0 ***  [≅0; ≅0] | Not included | Not included |
| General Bodily Injuries (ref. No) | 1.07  [0.69; 1.63] | 0.42 **  [0.24; 0.76] | 1.19  [0.42; 3.00] | 2.10**  [1.24; 3.58] | Not included | 0.36 *  [0.15; 0.84] | Not included | Not included |
| Physical Disability (from injury) (ref. No) | 4.57 ***  [2.29; 9.55] | 6.22 **  [2.05; 18.84] | 3.53 °  [0.90; 14.27] | 4.42 **  [1.63; 13.89] | 2.94 **  [1.47; 6.04] | Not included | Not included | 3.07 *  [1.21; 8.51] |
| Inpatient care  (ref. No) | 2.41 ***  [1.91; 3.02] | 4.48 ***  [2.96; 6.76] | 2.68 ***  [1.79; 4.01] | 2.13 ***  [1.50; 3.04] | 1.98 ***  [1.53; 2.55] | 4.77 ***  [2.94; 7.75] | 1.75 *  [1.12; 2.70] | 1.54 *  [1.06; 2.22] |
| Outpatient care (ref. No) | 1.46 ***  [1.20; 1.78] | 1.31  [0.83; 2.08] | 1.55 *  [1.05; 2.28] | 1.41 **  [1.095; 1.845] | Not included | Not included | Not included | Not included |
| Outpatient care (times in the last 12 months) | 1.06 ***  [1.04; 1.09] | 1.09 **  [1.03; 1.17] | 1.04 °  [0.99; 1.10] | 1.08 ***  [1.04; 1.13] | 1.03 °  [1.00; 1.06] | Not included | Not included | 1.03 *  [1.01; 1.07] |
| **Health Habits** | | | | | | | | |
| Current smoking status (ref. No) | 1.22 *  [1.01; 1.46] | 1.82 **  [1.25; 2.67] | 1.16  [0.82; 1.65] | 0.98  [0.77; 1.25] | Not included | Not included | Not included | Not included |
| Past smoking status (ref. No) | 0.84  [0.68; 1.04] | 0.74  [0.52; 1.06] | 1.03  [0.69; 1.51] | 1.08  [0.75; 1.54] | Not included | Not included | Not included | Not included |
| Alcohol consumption (ref. Abstainer/Occasional) |  |  |  |  |  |  |  |  |
| Non-Heavy Drinker | 0.56 ***  [0.45; 0.68] | 0.38 **  [0.22; 0.66] | 0.56**  [0.37; 0.84] | 0.58 ***  [0.45; 0.74] | 0.73 **  [0.59; 0.90] | Not included | 0.77  [0.52; 1.13] | 0.68 **  [0.52; 0.89] |
| Infrequent Heavy Drinker | 0.40 ***  [0.29; 0.55] | 0.46 **  [0.28; 0.77] | 0.29 ***  [0.14; 0.53] | 0.80  [0.41; 1.50] | 0.57 **  [0.40; 0.81] | Not included | 0.37 **  [0.18; 0.73] | 0.74  [0.36; 1.47 |
| Frequent Heavy Drinker | 0.97  [0.50; 1.79] | 1.78  [0.72; 4.20] | 0.76  [0.15; 2.67] | 0.91  [0.16; 4.09] | 1.12  [0.53; 2.25] | Not included | 0.83  [0.22; 2.46] | 0.64  [0.13; 2.60] |
| **Physical Activity (ref. Inactive or low)** | | | | | | | | |
| Moderate physical activity | 0.80  [0.61; 1.05] | 0.42**  [0.22; 0.81] | 0.78  [0.48; 1.29] | 0.95  [0.66; 1.38] | Not included | 0.50 °  [0.25; 1.00] | Not included | Not included |
| High physical activity | 0.63 ***  [0.49; 0.81] | 0.34 ***  [0.19; 0.62] | 0.80  [0.51; 1.27] | 0.75  [0.54; 1.06] |  | 0.46 *  [0.24; 0.92] |  |  |
| Fruit or vegetable nutrition (ref. No) | 0.93  [0.73; 1.20] | 1.18  [0.69; 2.05] | 0.79  [0.49; 1.32] | 0.99  [0.72; 1.36] | Not included | Not included | Not included | Not included |
| **Chronic Conditions** | | | | | | | | |
| Arthritis (ref. No) | 1.66 ***  [1.34; 2.06] | 1.89 ***  [1.32; 2.70] | 1.77 **  [1.21; 2.57] | 2.48 ***  [1.67; 3.73] | Not included | Not included | Not included | 1.46 °  [0.97; 2.18] |
| Stroke (ref. No) | 4.03 ***  [1.80; 9.50] | 4.0 5 **  [1.53; 10.78] | 3.92 *  [1.13; 14.52] | 5.56  [0.85; 79.84] | Not included | Not included | Not included | Not included |
| Angina (ref. No) | 2.05 **  [1.20; 3.46] | 3.83 *  [1.22; 12.08] | 2.53*  [1.17; 5.39] | 1.44  [0.64; 3.17] | Not included | Not included | Not included | Not included |
| Diabetes (ref. No) | 2.12 ***  [1.53; 2.94] | 2.16 *  [1.15; 4.11] | 1.87 *  [1.09; 3.15] | 2.38 ***  [1.45; 3.94] | Not included | Not included | Not included | Not included |
| Lung disease (ref. No) | 1.83 **  [1.17; 2.84] | 2.22  [0.70; 7.07] | 2.41 *  [1.18; 4.86] | 1.30  [0.69; 2.39] | Not included | Not included | Not included | Not included |
| Asthma (ref. No) | 0.89  [0.63; 1.23] | 1.34  [0.75; 2.39] | 0.58°  [0.29; 1.06] | 0.97  [0.59; 1.57] | Not included | Not included | 0.488 °  [0.22; 0.97] | Not included |
| Depression (ref. No) | 2.49 ***  [1.93; 3.20] | 4.87 ***  [3.02; 7.86] | 1.81 *  [1.10; 2.94] | 2.19 ***  [1.54; 3.12] | 1.38 *  [1.03; 1.85] | 2.46 **  [1.36; 4.46] | Not included | Not included |
| Hypertension (ref. No) | 1.58 ***  [1.28; 1.95] | 2.28 **  [1.43; 3.65] | 1.57 *  [1.07; 2.29] | 1.75 ***  [1.27; 2.43] | Not included | Not included | Not included | Not included |
| **Social Network** | | | | | | | | |
| Social Network index | 0.993  [0.986; 1.001] | 0.955 ***  [0.933; 0.978] | 0.984 *  [0.969; 0.999] | 0.988 *  [0.977; 0.998] | Not included | Not included | Not included | Not included |
| **Build Environment Assessment** | | | | | | | | |
| Reachability and usability of the neighborhood environment | 0.999  [0.994; 1.003] | 1.000  [0.990; 1.010] | 0.992 *  [0.985; 0.999] | 0.997  [0.991; 1.004] | Not included | Not included | 0.993°  [0.98; 1.00] | Not included |
| Hindrance of walkable environment | 1.007 **  [1.002; 1.012] | 1.014  [0.994; 1.034] | 1.004  [0.996; 1.012] | 1.010 **  [1.004; 1.017] | Not included | Not included | Not included | Not included |
| Open-to-public buildings, places and facilities | 1.001  [0.996; 1.006] | 0.979 *  [0.964; 0.995] | 0.999  [0.991; 1.007] | 0.995  [0.987; 1.002] | Not included | Not included | Not included | Not included |
| Usability of the living place/home | 0.992 ***  [0.987; 0.996] | 0.971 ***  [0.958; 0.985] | 0.994  [0.987; 1.001] | 0.985 ***  [0.977; 0.994] | Not included | Not included | Not included | 0.992 °  [0.984; 1.001] |

Notes. OR = Odds ratio, 95% CI = 95% Confidence Interval, p = p-value. ***= p < 0.001, ** = 0.01< p <0.001, * = 0.05 < p< 0.01, ° = 0.05 < p < 0.10. DCS pseudo- R^2^ = Design-adjusted Cox-Snell pseudo-R^2^, dAIC = Design-adjusted Akaike Information Criterion [46]
